# Comprehensive feedback on trainee surgeons’ non-technical skills

**DOI:** 10.5116/ijme.54b4.2196

**Published:** 2015-01-20

**Authors:** Lene Spanager, Peter Dieckmann, Randi Beier-Holgersen, Jacob Rosenberg, Doris Oestergaard

**Affiliations:** 1 Danish Institute for Medical Simulation, Capital Region of Denmark, Denmark; 1 Department of Surgery, Hilleroed Hospital, Denmark; 1 Department of Surgery, Herlev Hospital, Denmark

**Keywords:** feedback, surgery, communication, decision making, leadership

## Abstract

**Objectives:**

This study aimed to explore the content of conversations, feedback style, and perceived usefulness of feedback to trainee surgeons when conversations were stimulated by a tool for assessing surgeons’ non-technical skills.

**Methods:**

Trainee surgeons and their supervisors used the Non-Technical Skills for Surgeons in Denmark tool to stimulate feedback conversations. Audio recordings of post-operation feedback conversations were collected. Trainees and supervisors provided questionnaire responses on the usefulness and comprehensiveness of the feedback. The feedback conversations were qualitatively analyzed for content and feedback style. Usefulness was investigated using a scale from 1 to 5 and written comments were qualitatively analyzed.

**Results:**

Six trainees and six supervisors participated in eight feedback conversations. Eighty questionnaires (response rate 83 percent) were collected from 13 trainees and 12 supervisors. Conversations lasted median eight (2-15) minutes. Supervisors used the elements and categories in the tool to structure the content of the conversations. Supervisors tended to talk about the trainees’ actions and their own frames rather than attempting to understand the trainees’ perceptions. Supervisors and trainees welcomed the feedback opportunity and agreed that the conversations were useful and comprehensive.

**Conclusions:**

The content of the feedback conversations reflected the contents of the tool and the feedback was considered useful and comprehensive. However, supervisors talked primarily about their own frames, so in order for the feedback to reach its full potential, supervisors may benefit from training techniques to stimulate a deeper reflection among trainees.

## Introduction

Medical education often relies on formative assessment of learners, which aims to guide future learning, promote reflection, and shape values and behaviors. This is in line with the assessment for learning paradigm^1^ and will, ideally, stimulate life-long learning. Learners should be aware of what they should continue doing and what they should do better. Because self-assessment is largely unreliable,^2^ external feedback has been recognized as being essential for learning.^3^ Feedback from various sources helps participants shape their knowledge, skills, and attitudes.

Feedback has been defined as the provision of “specific information about the comparison between a trainee’s observed performance and a standard, with the intent to improve the trainee’s performance”.^4^ Learners acknowledge that feedback is important for fostering learning.^3^ Feedback leads to deeper learning by bridging the gap between experiencing an event and making sense of it.^5^

Feedback can be delivered in many forms: from the corrective and instructive to the more collaborative.^6^ Although the former is more common, current opinion argues that deeper learning requires feedback to be a dialogue; that is, the learner must be active and have his or her considerations sought and valued as part of the conversation.^7^ Other features of effective feedback include basing the feedback on direct observation, using specific, neutral language and establishing a respectful learning environment.^8^ Many influences on the interaction between supervisor and learner must be understood and taken into account in order for feedback to foster learning.^9^ For example, one study has indicated that feedback does not always address the concerns that supervisors have, and that feedback given to learners was more positive than the supervisor’s actual opinion of the learner.^10^

Studies have shown that feedback in a surgical setting improves technical performance as measured by economy of movement and feedback reduces the time required to complete the procedure.^11^^,^^12^ Feedback also improves safety parameters by reducing error rates.^11^ It has also been shown that postoperative video feedback after small bowel anastomosis ensured clinically relevant learning by reducing adverse events such as tearing of mesentery or intestinal serosa.^13^

Non-technical skills, such as decision making, leadership and communication, are trainable^14^^,^^15^  and are essential for conducting safe and efficient surgery. Effective learning of non-technical skills also depends on feedback. Although there are tools for guiding feedback on non-technical skills, most research to date has focused on the psychometrics of the tools; that is, ensuring valid and reliable assessments.^16^^-^^18^

However, feedback is the crucial element in the clinical use of the tools. Little has been written about the provision of feedback on the non-technical aspects of surgery. The tools can lose their value if the feedback from their use is not optimized. The present study aimed to address this gap by studying feedback on non-technical skills in a clinical setting and was guided by three specific research questions:

1)    What characterizes the content of feedback conversations regarding trainee surgeons’ non-technical skills when stimulated by a tool?

2)    What characterizes feedback conversations regarding trainee surgeons’ non-technical skills in terms of feedback style used?

3)    How do trainee surgeons and their supervisors perceive the usefulness of the feedback stimulated by a tool?

## Methods

This was an exploratory study that investigated feedback regarding trainee surgeons’ non-technical skills. Data were collected from two sources: feedback conversations and questionnaire responses. We constructed the questionnaire to explore the usefulness and the comprehensiveness of the feedback and to explore contextual factors such as time pressure and the perceived difficulty of the operation. As the questionnaire contained relatively simple questions, it was pilot-tested for understanding on a single operation. The pilot testers easily understood the questions, so no changes were made, and these data were not included in the analysis. 

### Setting

The study was conducted in 2013 in the capital region of Denmark at a university hospital that has two general surgical departments at different locations but with the same head. Surgical training in Denmark starts with a foundation year, followed by an introductory year to the specialty and then by five years’ specialty training (residency). Trainees can supplement their surgical experience by additional work in surgical departments between periods of employment in their formal training. The specialist education is modelled on the seven CanMEDS roles, assessments are formative and the available assessment tools predominantly focus on the medical expert role.^19^

### Participants

We used a convenience sample of surgical trainees and their supervisors, with the intention of achieving variety in both trainee level and in supervisor gender and surgical experience. Participants were recruited on a day-to-day basis when eligible procedures were available (that is, procedures in which a trainee was the main operator, but performed under the supervision of a senior colleague). Study participants received brief written and oral information about the study (purpose, observation and recording of conversations).

### Procedure

To stimulate feedback, participants were given the NOTSSdk (Non-Technical Skills for Surgeons in Denmark) assessment tool, which is based on the NOTSS developed in Scotland^20^ and adapted for the Danish healthcare system and culture. NOTSSdk is designed to provide trainees with post-operation feedback on their non-technical skills^21^ and consists of four categories: situation awareness, decision making, leadership, and communication & teamwork. Each of the four categories is described by three or four elements (Table 1), illustrated by numerous examples of good and poor behavior to guide the observation. The tool contains a scale with which to assess trainee performance, ranging from 1 to 5, where 1 is very poor performance and 5 is very good performance.

The psychometric properties of NOTSSdk were explored in a study using constructed simulation videos displaying real operating room (OR) teams performing an operation; this showed that the tool could be used with high inter-rater reliability.^22^ A study of assessment of real operations showed that it was sufficient to assess a trainee during five cases to gain reliable assessments using NOTSSdk.^23^ Participants were given the NOTSSdk user guide^24^ to familiarize themselves with the concepts, structure, and scale of the tool, and guidelines on its use for feedback before the study commenced. The first author (L.S.) was present in the operating room (OR) and observed the

operations to facilitate interpretation of the subsequent conversations. After the operation, the supervisor completed a hard copy of the NOTSSdk assessment form. The supervisor and trainee then had a feedback conversation alone in relatively undisturbed surroundings in the OR.

The conversations were audio-recorded and data gathering continued until saturation (that is, the point at which conversations contained no new information regarding the content of the feedback); this was obtained after eight observations. Subsequently, the trainee and supervisor completed a hard-copy questionnaire. Gathering of questionnaire data involved 40 surgical procedures and was collected along with the completed NOTSSdk assessment forms in a study exploring the reliability of assessments.^23^

**Table 1.** The NOTSSdk tool showing categories and elements

Category	Element
Situation awareness	Gathering information Understanding information Predicting and thinking ahead Monitoring own performance
Decision making	Considering options Selecting and communicating decisions Implementing and assessing decisions
Communication and teamwork	Exchanging information Establishing a shared understanding Coordinating activities
Leadership	Setting and maintaining standards Supporting others Coping with pressure

### Analysis

To explore the content of the feedback conversations (research question 1), all recordings were transcribed verbatim and read through once to obtain an overall impression of the content. Each transcript was then coded by L.S., who identified pieces of text containing information on one aspect of non-technical skills using directed qualitative content analysis.^25^  This is a qualitative analysis method that begins with a theory or relevant research findings (in this study, the categories / elements of NOTSSdk) to guide the initial coding. The interview pieces were then paraphrased and sorted according to the NOTSSdk elements, guided by the NOTSSdk definitions. Some pieces of the conversations clearly referred to non-technical skills, but were not a perfect match with the NOTSSdk elements. These were gathered separately and commented on. This analysis was then discussed with P.D. and D.O. and disagreements were resolved by consensus.

The ‘frames, actions, results’ model presented by Rudolph et al.^5^ was used to address research question 2 regarding the feedback style. The model was developed for simulation instructors and builds on theories on reflective practice; its central idea is that people make sense of the world through internal cognitive frames. The model states that peoples’ internal frames drive their actions, which have consequences. Good debriefing should elicit the trainees’ frames in order to understand their actions and ultimately potentially change frames. Once the frames are changed, actions will change too. We therefore assumed that a good feedback conversation would include discussion of the trainee’s actions and the trainee’s frames. L.S. and P.D. first coded three interviews independently using the five following codes: “supervisor frames” (when the supervisor’s talk provided insight to his/her internal frames used to make sense of the environment); “trainee frames” (trainee’s internal frames); “trainee actions” (trainee’s observable behavior or non-behavior); “supervisor actions” (supervisor’s observable behavior or non-behavior); and “results” (observable behavior or states in the team or the patient that were considered to have been prompted by actions or non-actions of the trainee). Discrepancies regarding which pieces of text to code were resolved by discussing and reaching agreement. It was decided that only meaningful sentences would be coded and not utterances such as “yes” and “no”. No changes were made to the coding structure. L.S. coded the last five conversations, counted the codes and transformed them into frequencies. The entire process can be described as a summative content analysis.^25^

The questionnaire ratings were counted for supervisors and trainees separately. Written comments were categorized and analyzed using qualitative content analysis of emergent themes.

We obtained oral and written informed consent from each participant and participants were informed that they could withdraw from the study at any time. Feedback conversations and questionnaire responses were anonymized upon transcription. The ethics committee in the Capital Region of Denmark stated that according to Danish law, the study was exempt from ethical approval, since it did not involve biomedical research (journal number: H-2-2012-FSP55).

## Results  

Data comprised eight feedback conversations and 80 questionnaires (40 from supervisors and 40 from trainees, response rate 83 percent).

Participants in the feedback conversations were two  female and four male trainees; one female and one male trainee participated twice. The median age of the participants was 31 (31–33) and their positions were introductory year to specialty training year 1–3. Participants had performed median two (0–30) independent operations of the same type, for which they received feedback, and median nine (1–30) supervised operations of the same kind. Supervisors were three female surgeons and three male surgeons, with two female surgeons providing feedback twice. The median supervisors’ age was 41 (35–50) and their positions were: one specialty training year 5, four specialty doctors, and one consultant. The operations were four laparoscopiccholecystectomies, two inguinal hernia repairs, one umbilical hernia repair and one pilonidal cyst excision. Questionnaires were returned by 13 trainees (specialty training year 1–4) and 12 supervisors (five senior residents, five specialty doctors and two consultants).

### Content of conversations

The conversations lasted median eight (2–15) minutes and were exclusively related to the trainees’ non-technical skills. The supervisors used all the categories in NOTSSdk and typically structured the conversation around the elements, addressing them one by one in the order they were written. Most feedback was congruent with the definitions and behavioral examples in NOTSSdk; exceptions were the inclusion of the patient as a co-decision maker and a team member to inform. In half of the conversations the supervisors regarded the elements “supporting others” and “coping with pressure” as irrelevant. Table 2 highlights issues from the conversations that differed from the definitions and behavioral examples in NOTSSdk.

**Table 2.** Issues discussed in feedback conversations that are not specifically contained in NOTSSdk. The quotations are shown for the elements in which they were mentioned in the conversations.

Category	Element	Quotation	Paraphrase	Comments
Situation awareness	Gathering information	*“I think it was nice that you knew the operative list the day before yesterday, and had considered which operations you would like to perform and where you have gotten to in your training.”* S*1, C^†^1	Knowing the operative list and knowing what one wants to do.	This takes place before arrival in the OR.
		*“And reading the patient chart before saying goodnight to the patient, so that you know what you are dealing with and take responsibility for. Not everyone necessarily does that… Sometimes things are a bit hasty.”* S*1, C^†^8	Taking responsibility for having gathered sufficient information.	The responsibility part is not specifically mentioned in NOTSSdk.
	Understanding information			
	Predicting and thinking ahead	*“And you cannot assume that others have read the operative indication.”* S*1, C^†^1	Must not assume that others have read the medical record.	This is potentially not observable.
	Monitoring own performance	*“I think you are a bit hard on yourself. Not that you shouldn’t be a perfectionist as a surgeon – of course you should. But I don’t see the reason for saying ‘oh this was not good.”* S*1, C^†^1	Appearing too self-critical – should be balanced with self-praise	Not specifically mentioned. NOTSSdk has a focus on not being too self-confident
Decision making	Considering options	*“I think that you are good at mentally adjusting smoothly. You show this several times when things don’t look as they usually do and things were a bit challenging.”* S*1, C^†^1	Smoothly adjusting to new situations.	Implicitly overlapping with situation awareness, but not specifically mentioned in NOTSSdk.
		*“The patient was also involved in deciding his positioning on the operating table. You complied with that and I think that was also good.”* S*6, C^†^6	Taking the patient’s wishes into consideration.	The patient is not part of the team in NOTSSdk.
	Selecting and communicating decisions	*“I think you could have made the decision, but it ends up being me that makes the decision.”* S*1, C^†^1	Letting the supervisor make the decisions.	The issue with supervised operations is not contained in NOTSSdk.
	Implementing and assessing decisions	*“Obviously, when I cannot hear your decision, then it is difficult to judge when you implement it and when you re-asses it.”* S*2, C^†^2	Unclear implementation and re-assessment of decisions.	Not specifically mentioned, but obviously problematic.
Communication and teamwork	Exchanging information	*“And you informed the patient so that he was extremely calm.”* S*6, C^†^6	Thoroughly informing the patient.	The patient is not part of the team in NOTSSdk.
		*“But you were more modest in your statements and that was probably the way it should be.”* S*6, C^†^6	Speaking politely and modestly.	NOTSSdk uses slightly less value-laden terms, but it could be included.
	Establishing a shared understanding	*“You manage to say in a good way that you haven’t sewed intracutaneously that often, so it is probably going to take a while […] That this is something you want to learn and that it is important. This is about establishing a shared understanding, right? That you make us want to teach you to sew intracutaneously.”* S*1, C^†^1	Creating acceptance of the learning position and making others want to teach you.	The learning aspect is mentioned in “leadership” but not from the trainee’s angle.
		*“I also think that you do not need to be oppositional and set limits if you are proficient. You need that if you are not skilled.”* S*1, C^†^1	Not having to assert oneself.	Used more subtly than the explicit poor examples of losing temper in NOTSSdk.
	Coordinating activities			
Leadership	Setting and maintaining standards	*“I think that you very explicit. You are really good, because you are explicit and communicate clearly and people relate to you. That is extremely nice, because you don’t come creeping in.”* S*5, C^†^5	Appearing prominent and looking people in the eyes.	Not mentioned specifically, but could potentially help build confidence in the leader.
	Supporting others	*“Or with the nurse anaesthetists: talk to them so they can see that you are in charge. That is what I mean by leading; it is very, very important that you support others”* S*2, C^†^2	Communicating so that the team can feel confident in the leader.	Not mentioned specifically.
	Coping with pressure			

* Supervisor; † Conversation

### Feedback style

We found the following opening styles for the conversations: (1) praising the trainee, (2) managing the trainee’s expectations, and (3) soliciting the trainee’s performance self-assessment. Criteria for the evaluation of the trainee’s performance were stated as (1) comparing the trainee’s performance with that of a skilled, specialist surgeon; (2) comparing it with that of peers; or (3) unclear. In none of the conversations did the supervisors provide a timeframe for the duration of the conversation.  Supervisors ended conversations either by checking that the trainee had understood the feedback or by reinforcing the good performance observed. Only one conversation ended with the trainee being set personal learning goals; in this case, the goals were set by the supervisor. Concerning the “frames, actions, results” codes we found that despite variation between conversations, the majority of the conversations concerned the trainees’ actions and the supervisors’ frames. Across the conversations, supervisor frames comprised median 47 (23–57) percent of the conversations (as expressed by count of codes); trainee frames represented median 20 (10–25) percent, trainee actions median 28 (20–50) percent, supervisor actions median one (0–9) percent, and results median five (0–9) percent.

### Usefulness

Ratings for usefulness and comprehensiveness of the feedback were above average/high for both trainees and supervisors (Table 3), whereas ratings varied more for the contextual factors “time pressure involved in the feedback” and “difficulty of the operation”. Participants’ comments indicated that the tool directed their attention to issues not usually covered in feedback and provided the occasion and the structure for a neutral and systematic approach (Table 4). Challenges mentioned centered on learning a new concept (non-technical skills) and acquiring a new method (feedback using NOTSSdk).

**Table 3.** Questionnaire responses regarding the usefulness of NOTSSdk feedback*

Item	Trainees (n=40)	Supervisors (n=40)
	Median (range)	Median (range)
It was useful for me to get feedback on my non-technical skills using NOTSSdk (for trainees) *or* It was useful for my teaching to use NOTSSdk for feedback on the trainees’ non-technical skills (for supervisors)	4.0 (3-5)	4.0 (3-5)
The relevant points were covered in the feedback	4.0 (3-5)	3.5 (2-5)
There was time pressure for this feedback session	3.0 (1-5)	3.0 (1-5)
This operation was difficult for me/the trainee	3.0 (1-5)	3.0 (1-5)

* Scale: 1= strongly disagree; 2= disagree; 3= neutral; 4=agree; 5=strongly agree.

**Table 4.** Categories of comments on the usefulness and challenges of using NOTSSdk for feedback

Trainee answers	Supervisor answers
In what aspects did NOTSSdk help you to provide / get feedback?	
· Raises awareness of the importance of communicating one’s thoughts, especially during new procedures	· Creates the necessary room and occasion for feedback
· Usable to go over the procedure before starting	· Directs attention to issues not usually covered in feedback
· Highlights skills that are rarely in focus (i.e. the role as a leader)	· Provides structure for the conversation, making it less abstract and making the supervisor more neutral
· Provides a scheme for a systematic approach	· Stimulates self-reflection
· Brings constructive feedback and puts focus on things you are not that good at	· Hopefully results in better- skilled surgeons
What were the challenges in using NOTSSdk for the feedback?	
· Some elements are not relevant for small procedures	· Extensive process if the procedure is small or uncomplicated
· Different mind-set that requires familiarization	· Novel concept and new method
· Too little time	· To place the issue in the right category and assess what is relevant
· Difficult to focus on the non-technical aspects when the procedure is new	· Too little time
· Difficult to formulate these softer values	· Potential assessment bias if you know the trainee well
Anything else you would have liked to provide / get feedback on?	
	· The trainee’s technical skills, but the occasion was used to provide feedback on that too

## Discussion

This study showed that, with minimal introduction, surgeons could use NOTSSdk to provide feedback on trainee surgeons’ non-technical performance. Although the feedback was useful and comprehensive, conversations uncovered more of the supervisors’ own internal frames rather than discussing trainee frames and the results of their actions.

### Content of conversations and use of NOTSSdk  

The content of the conversations revealed substantial overlap with the definitions and behavioral examples provided in NOTSSdk. This indicates that the tool provides a terminology and language relevant to the supervisors that can easily be applied in feedback conversations. We identified a broader use of the non-technical skills terminology compared with that in the NOTSSdk user guide; for example, by including the patient in the team. Patients was deliberately excluded in NOTSSdk in order to keep the tool focused on observable behavior during the phase of surgical treatment occurring in the OR, during which the patient is anesthetized most of the time. However, users’ interpretation of the terms in their own way facilitates ownership of the tool, and focusing on the patient is the ultimate intention of good non-technical skills. The supervisors in this study focused on future lessons for the trainee as well as feedback based on their observations. As the behavioral examples in NOTSSdk are largely based on observable behavior, this is a natural explanation for the slightly broader use of the terminology in the feedback conversations. Another explanation could be that supervisors provided their own opinions on what constitutes good non-technical performance, thereby interpreting the concepts more broadly.

The conversations revealed that supervisors regularly regarded the elements “supporting others” and “coping with pressure” as irrelevant. This might be because the operations did not put any pressure on the trainee or require the support of team members, due to the elective, supervised sample of procedures in this study. Another explanation could be that supervisors had a different understanding of their job and responsibilities during an operation and might not have been fully aware of the influence that the elements had on patient safety and teamwork. Supporting others covers providing mental and physical support to team members by supervising and motivating, by inviting questions and by establishing a professional atmosphere; it could be argued that this is relevant irrespective of procedure’s complexity. An observational study of surgeons’ leadership behaviors in the OR reported that although surgeons were engaged in both task and team maintenance functions, the guiding and supporting behaviors observed were related to task accomplishment rather than team building.^26^ This supports the findings from the present study and indicates that surgeons could focus more on the leadership skills involving motivating and enabling team members.

### Feedback style

Although feedback and debriefing are often used interchangeably, there are differences between the concepts. Feedback focuses on the information transfer between individuals, whereas debriefing in simulation can be seen as a “social practice during which people purposely interact with each other and the environment, reflecting on the common experience they had during the scenario.”^27^ This highlights that debriefing encompasses feedback, but emphasizes reflection as a means to stimulate deeper learning. Models exist for teaching and debriefing in the OR,^28^ in the simulated setting,^29^ and for both.^30^ Reinforcement, open questions, and corrective suggestions given in a respectful manner appeared in the conversations; however, the feedback conversations were not ideal when compared with the above-mentioned models, as it was usually the supervisors’ frames that surfaced. Some supervisors offered general advice on what to do in a similar situation and some extrapolated more concretely to other operations. However, many comments were quite loosely related to the concrete actions of the trainee. In this sense, some of the feedback conversations did not follow the guidelines for effective feedback mentioned in the introduction.^9^ Greater attempts to understand trainees’ perceptions of the operation and the mental models behind their actions might enable the trainees to form their own thoughts,^28^ thereby stimulating deeper learning.

We found a few attempts, by either trainees or supervisors, to formulate personal learning goals. This is otherwise desirable to consolidate learning ^6^ and would be accordant with the state of the art in post-simulation debriefings.^31^ The learning goals could be set before the operation in order to focus the learning on specific outcomes during the procedure; alternatively, the learning goals could be set during the feedback conversation to guide future efforts. Nonetheless, it is unsurprising that the supervisors in this study did not try harder to understand the trainees’ frames, as even experienced simulation instructors have difficulties posing open questions and instead tend to supply their own opinion.^27^ This is also in line with a study on reflection levels.^32^ Considering that simulation courses provide protected time and a structured framework for feedback, it is understandable that the picture in clinical practice is no better. 

### Usefulness

Trainees reported that the feedback they received was useful and comprehensive, although there was still room for improvement, as ratings ranged from 3 to 5. This could reflect the feedback not always being considered sufficiently content specific in the short conversations, or it could reflect that the feedback style tended to be supervisor-driven, rendering the trainee passive, rather than being given on trainee demand.^33^ We have anecdotal evidence that the study was an intervention in itself. Both trainees and trainers reported that feedback is usually given intra-operatively in an informal way and typically only relates to technical skills. This is in line with the findings of a study on surgeons’ debriefing practices and deviations from the ideal debriefing.^34^

Both trainees and supervisors mentioned time as a barrier to optimal feedback. This is an inherent factor in busy clinical life, but its importance in this study was contradicted by the somewhat short duration of the conversations (2–15 minutes) and by the perceived time pressure, which was rated as ‘neutral’ by both trainees and supervisors.

This study has certain limitations. Being a small study with few participants conducted at only one institution, with saturation steered by the content of the conversations, it is likely that we did not cover all feedback styles that surgeons can display.  Furthermore, the study was susceptible to recruitment bias, possibly including supervisors with a positive attitude to feedback and non-technical aspects of surgery. However, the study aimed to describe practice, and data were sufficient to hypothesize that there are different feedback styles. Increasing the sample size might have revealed more patterns or provided information about the distribution of the different styles. This study was an explorative study that did not involve any comparison. As anecdotal evidence informed us that feedback on non-technical skills is rarely provided, we found it futile to aim for baseline measurements. Moreover, as the scope of this study was to explore the applicability of NOTSSdk, we made no attempts to formally assess the quality of the feedback. Accordingly, we cannot reach any conclusions about which feedback style is the most effective. This would be worth investigating in future studies. The present study was conducted in a clinical setting and the conversations were framed as feedback conversations rather than debriefings. Accordingly, it might not be appropriate to judge the conversations against debriefing practices. 

Regarding the implications of this study, we found that the supervisors who engaged in feedback conversations concerning their trainees’ non-technical skills—a practice they were unfamiliar with—welcomed the feedback opportunity, as did trainees. This is encouraging in terms of implementing regular feedback on trainees’ non-technical skills, and is likely to be equally relevant in specialties other than surgery. The results suggest that, in particular, greater focus could be placed on developing leadership behaviors during medical education. In our view, the generalizable outcome of our study was the result that feedback conversations are usable and feasible. However, more time for supervisors to familiarize themselves with the concept, the methods, and the vocabulary pertaining to non-technical skills could make the feedback even more useful for trainees. Expert-derived guidelines recommend that faculty involved in assessment of non-technical skills (which precedes the feedback given) are trained to ensure high quality of assessments.^35^ For full impact of the feedback in terms of stimulating deeper trainee reflection, such training would ideally involve peer discussions and practicing techniques for creating deeper reflection. A major needed next step in surgery is making feedback part of the institutional culture, addressing both technical and non-technical skills.

## Conclusion

This study showed that feedback conversations on trainee surgeons’ non-technical skills were characterized by a content close to the contents of the tool (NOTSSdk) used, but also with supervisors’ own interpretations, possibly facilitating ownership. The conversations revealed more of the supervisors’ frames than the trainees’ frames, which suggests that supervisors would benefit from practicing techniques for stimulating reflective practice in their trainees. Although a new mindset is required, supervisors and trainees found the conversations useful and comprehensive.

## 

### Acknowledgements

The authors wish to thank Christine Hangaard for help with the distribution and collection of questionnaires.

### Conflict of Interest

The authors declare that they have no conflict of interest.
